# Bi-Chloride of Mercury Baths in the Treatment of Variola

**Published:** 1899-11

**Authors:** R. H. L. Bibb

**Affiliations:** Chief Surgeon to the Mexican National Railroad; Late Physician and Surgeon in Chief to the American Hospital, City of Mexico, Etc., Etc.


For Texas Medical Journal.

### Bi-Chloride of Mercury Baths in the Treatment of Variola.

BY R. H. L. BIBB, M. D.,
Chief Surgeon to the Mexican National Railroad; Late Physician and Surgeon in Chief to the American Hospital, City of Mexico, etc., etc.

Some ten years ago my distinguished friend, Dr. T. C. Osborn, of Cleburne, Texas,—to whom I beg, thus publicly, to tender, most cordially, most heartily, expressions of deepest gratitude for this invaluable contribution to the cause of suffering humanity—wrote me, in laudatory terms, of the potency of bi-chloride of mercury baths in the treatment of variola, which he had employed for many years, and for which he claimed most marvelous results, urging me to lose no opportunity to accord the benefits of its puissance to such of my patients as might fall victims to this pestilential malady.

Dr. Osborn assured me that by properly, timely and frequently sponging a variolous patient—from head to foot—with a warm, as hot as the patient could bear, solution of bi-chloride of mercury in distilled water, one to one thousand and stronger, that the disease could be arrested before reaching the suppurative stage; that the intolerable itching and the insufferable odor, so prominent in smallpox, would be avoided; that pitting, with its disfiguration, and suppuration with its many dangers would be entirely prevented, and that desquamation, with its scabs and its scales, being rendered aseptic, would no longer be a menace to homes and to communities.

For a long time—much to my regret now—notwithstanding their high and authoritative source, I regarded these claims with extreme skepticism, for I believed that the doctor, although a physician of ripe and varied experience, and an observer of acute perception and much erudition, had attributed abortive cases of variola to the result of his treatment; and it was not until I took charge, as physician and surgeon in chief, to the American Hospital in the City of Mexico, in January, 1897, did I have the temerity to try Dr. Osborn's treatment, although I had many cases of smallpox in the meantime, and had never forgotten his eloquent appeals for its trial, nor his earnest assurances of its efficacy.

A few days after the date last written—I write from memory—I ordered a young Frenchman, aged 22 years, to the smallpox wards of the American Hospital, with one of the most typical, and at the same time one of the most violent, unmanageable and unpromising cases of smallpox that ever came under my observation;—so violent, unmanageable and wildy delirious was the patient that I was forced to order him the constant attention of two special male nurses, who, in order to control him, were obliged to strap him, hands and feet, to the bed. The disease was in the papular stage, the papules literally covering the entire body, including the eyes, ears, nose, mouth and throat. He could swallow absolutely nothing. His temperature was high, his pulse was rapid and feeble, and his general condition indicative of little hope.

Believing the young man doomed; that no treatment would save him, I determined to give Dr. Osborn's treatment the benefit of the patient, should it fail to be of service to him, as I fully expected it would. Accordingly he was ordered to be liberally sponged over the entire body with a warm solution of bi-chloride of mercury, 1 to

500 in distilled water, every four hours; two centigrammes of sulphate of morphia, hypodermically p. r. n.; his eyes, nose, mouth and throat irrigated with a warm saturated solution of boric acid, in distilled water, also, every four hours, and a nutritive clyster of two hundred grammes of peptonized milk and egg, with thirty grammes of brandy and fifteen drops of laudanum, every six hours.

At my regular morning visit, forty-eight hours after the treatment outlined above had been instituted, and which had been most faithfully and scrupulously administered, the nurse in charge, an old experienced smallpox nurse, informed me that while the patient was free from fever, had slept and had been quiet and rational from the night before, it not being necessary to restrain him longer, he thought he was worse in that the vesicles were shrivelling up and turning black, as if the patient were afflicted with *"variola hemorrhagica,"* as he had never seen this phenomenon in any other form of the disease, although he had nursed many hundred cases of smallpox, both in natives and in foreigners.

From a careful examination of the patient's general condition, the great improvement, as compared with his condition on the previous evening, the complete absence of fever, of nervous symptoms and of hemorrhage from the kidneys, nose and other mucous membranes, I could easily, and did exclude "variola hemorrhagica." This left me to decide whether the disease was aborting (as it sometimes does) or whether it was being favorably influenced by the treatment to which it had been subjected. I adopted the latter hypothesis, and as subsequent events and a more enlarged experience demonstrated, chose correctly, for my patient recovered rapidly, without itching, without smell, without pustulation, without secondary complications, and, excepting a few pits about the forehead, the nose and mouth, where the vesicles were ruptured and repeatedly irritated while cleaning his eyes, nose and mouth,—*without* pitting or other disfigurement.

This, in brief, outlines the treatment and results in forty cases of smallpox treated at the American Hospital while I was in charge of that institution, and of fifteen additional cases treated outside during that time and subsequent to my retirement therefrom.

In none of the fifty-five cases thus treated (I refer to the treatment wherein the bi-chloride of mercury baths, as detailed above, constituted *the essential* treatment addressed to the disease; of course, subsidiary treatment was used to combat individual symptoms in individual cases, such as correcting diarrhœa, etc., where treatment *antedated pustulation,* i. e., when it began with or before vesiculation) was there troublesome itching, pustulation, foul odors, pitting or secondary consequences of any character whatever—all heretofore much dreaded accompaniments of smallpox; and, in but one case, a female patient who entered the hospital with pustulation somewhat advanced, and who was treated with the bi-chloride in the form of blue tablets (I had always used red or white tablets), were there abscesses.

Of the fifty-five cases herein included, two died. One, a young man, of alcoholic and of syphilitic antecedents, 35 years of age, died of hæmaturia, in the papillary stage of the disease, twenty-four hours after admission. The other, an old hospital nurse, 73 years old, the subject of Bright's disease, died of heart failure, also in the papillary stage of his illness, a few hours after the first sponging. I will here state my conviction that the excessive fear excited in this patient by the knowledge that he had smallpox had much to do with his sudden death, if, indeed, it did not cause it directly.

Children and adults, male and female were treated alike; i. e., with a solution of the same concentration, one of bi-chloride to five hundred of distilled water, with alike happy results.

Stomatitis, which is frequent in variola, however treated, occurred but once in the fifty-five cases; and although the mercurial baths were continued, instead of increasing, as it most surely would have done had it been caused by them, improved *pari passu,* with improvement in the patient. Nothing was used against it except the boric acid solution already mentioned.

Unfortunately, the ill health of my family forced me to sever my connection with the American Hospital, and to leave the City of Mexico before I could determine, by experimental inoculation on calves, as I had arranged to, whether the scabs from patients with smallpox thus treated are sterile or not, as to the virus of that disease —one can easily imagine them to be sterile, and should, reasonably, expect them to be, the treatment having prevented pustulation,— as to the usual pyogenic organisms, when one remembered that these germs enter the vesicles, through their walls from without, and begin the pyogenic process, at first superficially, the deeper contents becoming subsequently involved, if it be admitted that suppuration is always due to their presence, unless it can be shown that the presence of these organisms does not, of necessity, imply the pyogenic process. The viability or not, of pus organisms, in the scabs, however, may be determined by culture experiments; that of the variola virus, whatever it may be, by inoculation experiments, subjects of sufficient importance, in my humble opinion, to challenge attention from expert bacteriologists—propositions to which I intend to address myself whenever a favorable opportunity presents.

The first change noted in the vesicles treated by the bi-chloride, as detailed above, is, that they become opalescent, as if their contained fluid had coagulated, and the surrounding zone of inflammation fades away, the vesicles speedily shrivelling up, their contents gradually turning black, so that by the time they are dry, they resemble small tacks driven into the skin, and with desquamation, they fall out, as one patient put it, "like peas from the pod."

A number of my confrères, among the foreign physicians in the City of Mexico, used the bi-chloride treatment with results equally (if not more) satisfactory as those obtained by myself. One of them, Dr. Schmidtlein, in order to contrast the results of the bi-chloride baths with the results from carbolic acid and from ichthyol, used the three substances on different parts of the body with effects immeasurably in favor of the former method.

The mortality attending this, the bi-chloride, method in my fifty-five cases, has been less than four per cent., while Dr. Lowry and Dr. Parsons, of the City of Mexico, whose experience with it now numbers several hundred cases, informed me last March that neither of them had lost a case since they began to use the bi-chloride baths in 1897, although they had treated young and old with it, confluent and discrete smallpox, hospital and private patients, natives and foreigners.

In my humble judgment, the foregoing facts warrant the conclusion:

First.—That the bi-chloride of mercury, applied as herein indicated and *soon enough,* is the rational treatment of variola.

Second.—That its use will prevent itching, foul odors, pustulation, abscesses and pitting.

Third.—That it will greatly lessen the mortality and suffering from one of the most loathsome diseases that affects the human race.

Fourth.—That it should be expected to destroy the variolous virus in the vesicles and pustules, thus rendering the scabs and scales harmless, reducing, thereby, to the minimum, the dangers of infection from a given case of smallpox.

Saltillo, Mexico, April 1, 1899.

NOTE.—This article was intended to be read at the last meeting of the Texas State Medical Association (San Antonio, April, 1899), but the author was prevented from attending. When he decided that he could not attend, it was too late to forward the paper before the Association adjourned. It has been secured for the JOURNAL, and is published now, opportunely, smallpox being reported in many localities in the State, in the hope that its readers will give the method a fair trial.—ED.

